# Long-Term Treatment Outcome of Progressive *Mycobacterium avium* Complex Pulmonary Disease

**DOI:** 10.3390/jcm9051315

**Published:** 2020-05-02

**Authors:** Kiyoharu Fukushima, Seigo Kitada, Yuko Abe, Yuji Yamamoto, Takanori Matsuki, Hiroyuki Kagawa, Yohei Oshitani, Kazuyuki Tsujino, Kenji Yoshimura, Mari Miki, Keisuke Miki, Hiroshi Kida

**Affiliations:** 1 Department of Respiratory Medicine, National Hospital Organization Osaka Toneyama Medical Centre, 5-1-1 Toneyama, Toyonaka, Osaka 560-8552, Japan; fukushima@imed3.med.osaka-u.ac.jp (K.F.); yamamoto.yuji.yf@mail.hosp.go.jp (Y.Y.); matsuki.takanori.qn@mail.hosp.go.jp (T.M.); kagawa.hiroyuki.kx@mail.hosp.go.jp (H.K.); oshitani.yohei.fp@mail.hosp.go.jp (Y.O.); tsujino.kazuyuki.bh@mail.hosp.go.jp (K.T.); yoshimura.kenji.ka@mail.hosp.go.jp (K.Y.); miki.mari.bk@mail.hosp.go.jp (M.M.); miki.keisuke.pu@mail.hosp.go.jp (K.M.); 2 Department of Respiratory Medicine and Clinical Immunology, Osaka University Graduate School of Medicine, 2-2 Yamadaoka, Suita, Osaka 565-0871, Japan; y.abe@imed3.med.osaka-u.ac.jp; 3 Department of Respiratory Medicine, Yao Tokusyuukai General Hospital, 1-17 Wakakusa-cho, Yao, Osaka 581-0011, Japan; kitadas1@mac.com

**Keywords:** *Mycobacterium avium*, *Mycobacterium intracellulare*, nodular bronchiectasis, non-tuberculous mycobacteria, pulmonary aspergillosis, rare pulmonary disease

## Abstract

Background: Multidrug therapy is essential for preventing respiratory failure in patients with highly progressive *Mycobacterium avium* complex pulmonary disease (MAC-PD). However, the prognosis and long-term outcome following combination therapy is poorly understood. Methods: We retrospectively evaluated the clinical characteristics and long-term outcomes in patients with chemo-naïve progressive MAC-PD, hospitalized for first-line multidrug therapy. Results: Among 125 patients, 86 (68.8%) received standardized treatment (rifampicin, ethambutol, clarithromycin), 25 (20.0%) received a fluoroquinolone (FQ)-containing regimen, and 53 (42.4%) received aminoglycoside injection. The sputum conversion rate was 80.0%, and was independently associated with standardized treatment. The incidence of refractory disease (45.6%) was independently and negatively associated with standardized regimen and aminoglycoside use. Choice of an FQ-containing regimen was not associated with positive outcome. Clarithromycin resistance occurred in 16.8% and was independently associated with refractory disease. MAC-PD-associated death occurred in 3.3% of patients with non-cavitary nodular bronchiectasis (NB) and 21.3% with cavitary MAC-PD over a median follow-up period of 56.4 months. The rates of MAC-PD-associated death were comparable between cavitary-NB and fibrocavitary disease. Concurrent chronic pulmonary aspergillosis (CPA) occurred in 13 (17.3%) patients with cavitary MAC-PD, and age, diabetes mellitus, and CPA were independent risk factors for mortality. Conclusions: Standardized intensive multidrug treatment reduces disease progression and persistence in progressive MAC-PD. Cavitary NB may differ from, rather than being just an advanced stage of, non-cavitary NB. The high incidence and significant mortality of CPA in cavitary MAC-PD highlight the need for early diagnosis and treatment.

## 1. Introduction

Non-tuberculous mycobacterial pulmonary disease (NTM-PD) has been increasingly implicated in a broad range of infectious diseases both in Japan and worldwide [1,2]. *Mycobacterium avium* complex (MAC), predominantly comprising *M. avium* and *M. intracellulare*, is the most common etiology of NTM lung disease [3]. The correct diagnosis and management of this disease are extremely important. Although, multidrug combination therapy is considered essential for preventing respiratory failure in highly progressive MAC-PD, and rifampicin (RFP) + ethambutol (EB) + macrolides are considered to be the standardized regimen, no large studies have validated the appropriateness of current treatment guidelines for MAC-PD [4,5,6]. Fluoroquinolones (FQ) have also reportedly been effective and represent a promising alternative for MAC infection [7,8]; however, data regarding combination therapy including FQ are lacking. Furthermore, the addition of an aminoglycoside may also be recommended in patients with severe and advanced disease, but their long term benefits also remain unproven [9]. There is thus a need to determine the efficacy and long-term outcome of combination therapy in patients with progressive MAC-PD. In this study, we aimed to evaluate the clinical outcomes of patients treated with combined antimicrobial drugs for progressive MAC-PD, and examine the factors associated with refractory disease and mortality.

## 2. Material and Methods

### 2.1. Study Design and Patients

This retrospective study was performed in accordance with the Declaration of Helsinki and was approved by the institutional research ethics board of the National Hospital Organization Osaka Toneyama Medical Centre (TNH-2019033), which waived the requirement for informed consent due to the retrospective nature of the analysis. The medical records of patients with non-tuberculous mycobacterial pulmonary disease (NTM-PD) hospitalized at the National Hospital Organization Osaka Toneyama Medical Centre between January 2012 and April 2018 were retrospectively reviewed. None of the patients tested positive for human immunodeficiency virus (HIV). None of the patients received clarithromycin (CAM) monotherapy before hospitalization. All patients were followed until their last visit, death, or the end of study period (31 October 2019).

### 2.2. Data Collection

Clinical data were collected from medical records. Baseline clinical parameters were obtained within 1 month of the initial diagnosis. Patient data included age, sex, body mass index (BMI), smoking status, underlying diseases, acquired comorbidities, treatment durations, antimicrobial treatment for MAC-PD, results of bacterial culture, and chest computed tomography (CT) findings. The diagnosis of chronic pulmonary aspergillosis (CPA) was based on the European Respiratory Society (ERS) and European Society of Clinical Microbiology and Infectious Diseases (ESCMID) guidelines for the management of CPA combined with clinical symptoms, radiological findings, positive *Aspergillus* serology, or isolation of *Aspergillus* species from respiratory samples [10].

### 2.3. Radiological Evaluation

Radiographic abnormalities were classified according to distinct disease patterns on chest CT. Patients with fibrocavitary lesions and pleural thickening mainly in the upper lobes on CT were diagnosed with fibrocavitary (FC) disease, and patients with multiple nodules on CT and bronchiectasis were diagnosed with nodular bronchiectatic (NB) disease. Patients with no specific pattern on CT, such as solitary pulmonary nodules, were diagnosed with unclassifiable disease.

### 2.4. Antibiotic Therapy and Treatment Outcomes

All patients who began daily antibiotic therapy received standardized (rifampicin (RFP) + ethambutol (EB) + CAM) or modified combination antibiotic therapy with CAM, RFP, EB, and fluoroquinolones (gatifloxacin, sitafloxacin, moxifloxacin, garenoxacin, or levofloxacin) [6]. Aminoglycoside antibiotics were administered intramuscularly (kanamycin and streptomycin) or intravenously (amikacin). Streptomycin and kanamycin were administered intramuscularly three times a week for the first several months, at the discretion of the attending physician. Amikacin was administered daily for 28 days, followed by kanamycin or streptomycin for the first several months. The main treatment regimen was evaluated for 3 months after treatment initiation.

### 2.5. Sputum Examination

Sputum cultures were examined for acid-fast bacilli using 2% Ogawa egg medium (Japan BCG, Tokyo, Japan) or a mycobacteria growth indicator tube (Japan Becton, Dickinson and Company, Tokyo Japan). Nontuberculous mycobacterial species were identified using the AccuProbe (Gen-Probe Inc., San Diego, CA, USA) or COBAS AMPLICOR (Roche Diagnostic, Tokyo, Japan) systems or by DNA−DNA hybridization assay (Kyokuto Pharmaceutical Industrial, Tokyo, Japan) CAM susceptibility was determined by broth microdilution (BrothMIC NTM; Kyokuto Pharmaceutical Industrial, Tokyo, Japan), and CAM resistance was defined as a minimum inhibitory concentration ≥32 µg/mL [11].

### 2.6. Definition of Sputum Conversion, Recurrence, and Refractory Case

Patient status at the end of follow-up was recorded in terms of deceased or alive, microbiologic or radiologic recurrence, and cause of death, if applicable. Sputum conversion was defined as more than three consecutive negative sputum cultures over a period of 3 months. In patients who achieved sputum conversion, clinical recurrence was defined by at least two positive sputum cultures. Refractory cases were those with no negative sputum conversion, or recurrent cases as sustained positive sputum culture until the end of follow-up.

### 2.7. Statistical Analysis

All statistical analyses were performed using GraphPad Prism version 7 (GraphPad Software, San Diego, CA, USA) and JMP Pro 13 (SAS Institute Inc., Cary, NC, USA). Continuous variables were reported as mean and standard deviation, or median and interquartile range. Patient groups were compared using the Mann–Whitney U test for continuous variables, and χ^2^ or Fisher’s exact test for categorical variables. Potential independent factors identified as significant by univariate analysis were evaluated by multivariate logistic regression analysis, in addition to age, sex, BMI, and cavity. Cumulative rates of MAC-PD-associated death were estimated using the Kaplan–Meier method and compared using log-rank tests. A two-sided *p* < 0.05 was considered significant.

## 3. Results

### 3.1. Patient Selection and Treatment Modalities

A total of 331 patients with a main diagnosis of NTM-PD (international classification of disease (ICD)-10 code) were hospitalized during the study period. Among 331 patients, 313 met the American Thoracic Society/Infectious Diseases Society of America criteria for non-tuberculous mycobacterial pulmonary disease [6], and 252 met the diagnostic criteria for MAC-PD. Among these, 125 chemo-naïve patients were hospitalized for the induction of first-line combination antibiotic therapy in our hospital (Figure S1). The treatments regimens are shown in Table 1. Of the 125 patients, 86 (68.8%) were treated with an RFP + EB + CAM-based regimen, 21 (16.8 %) were treated with EB + CAM with or without FQ, 18 (14.4 %) were treated with another treatment regimen (CAM + RFP, *n* = 4; CAM + RFP + FQ, *n* = 7; EB + RFP, *n* = 2; CAM + FQ, *n* = 4; EB + FQ, *n* = 1), and 25 (20.0%) were treated with an FQ-containing regimen (RFP + EB + CAM + FQ, *n* = 4; EB + CAM + FQ, *n* = 9; CAM + RFP + FQ, *n* = 7; CAM + FQ, *n* = 4; EB + FQ, *n* = 1). Aminoglycoside injection was used in 53 (42.4%). All 125 patients were tested for drug susceptibility before treatment. Susceptibility to CAM was as follows: sensitive (minimum inhibitory concentration (MIC) ≤ 2, *n* = 124), resistant (MIC > 32, *n* = 1). Susceptibility to levofloxacin was as follows: sensitive (MIC < 2, *n* = 84), intermediate (MIC ≥ 2 to < 8, *n* = 30), resistant (MIC ≥ 8, *n* = 11). Susceptibility to moxifloxacin (MFLX) was tested in five levofloxacin-resistant patients and all five strains were resistant to MFLX (MIC 4, *n* = 3; MIC > 8, *n* = 2). Susceptibility to aminoglycosides was as follows: amikacin: sensitive (MIC < 4, *n* = 17), intermediate (MIC ≥ 4 to <16, *n* = 82), resistant (MIC ≥ 16, *n* = 26); kanamycin: sensitive (MIC < 4, *n* = 15), intermediate (MIC ≥ 4 to <16, *n* = 83), resistant (MIC ≥ 16, *n* = 26); streptomycin: sensitive (MIC < 4, *n* = 56), intermediate (MIC ≥ 4 to <8, *n* = 36), resistant (MIC ≥ 8, *n* = 32).

### 3.2. Baseline Characteristics

The median age and BMI of the patients were comparable to previous reports [12]. The main underlying diseases were previous pulmonary tuberculosis (8.8%), chronic obstructive pulmonary disease (4.0%), and diabetes mellitus (6.4%). Of the bacterial species identified, *M. avium* was the most frequent, followed by *M. intracellulare* and both (Table 2).

### 3.3. Predictive Factors for Sputum Conversion

Of the 125 patients, 100 (80.0%) patients achieved sputum conversion. Univariate analysis identified diabetes mellitus as a risk factor for failure of sputum conversion, while a standardized (RFP + EB + CAM-based) regimen and aminoglycoside use were significantly associated with positive outcome. Multiple logistic regression analysis revealed that a standardized regimen (*p* = 0.0238) was significantly and independently associated with outcome (Table 2).

### 3.4. Risk Factors for Refractory Disease

Univariate analysis identified BMI, concurrent CPA, and acquired CAM resistance as risk factors for refractory disease, and standardized (RFP + EB + CAM-based) regimen and aminoglycoside use as significantly associated with positive outcome. Multiple logistic regression analysis revealed that BMI (*p* = 0.0162), standardized regimen (*p* = 0.0023), aminoglycoside use (*p* = 0.0056), and acquired CAM resistance (*p* = 0.0002) were independently associated with outcome (Table 3).

### 3.5. Analysis of FQ-Containing Regimens

FQ-containing regimens showed no additional benefit in terms of sputum conversion and preventing refractory disease (Table 2 and Table 3). We further evaluated FQs as potential candidates of multidrug regimens by comparing RFP + EB + CAM and RFP/EB + CAM + FQ regimens (Table 4). The baseline characteristics and initial sputum conversion rates of the two regimens were comparable. However, the number of refractory cases and acquired CAM resistance were significantly higher in patients using the RFP/EB + CAM + FQ regimen.

### 3.6. Mortality

Death from any cause occurred in 27 (21.6%) patients over a median follow-up period of 56.4 months (38.1–82.9) (Figure S2). Among these, 18 patients died from MAC-PD-associated causes (progression of MAC-PD, *n* = 14; respiratory failure due to MAC-PD, *n* = 2; concomitant bacterial or fungal infection, *n* = 2) and nine patients died from other causes (interstitial pneumonia, *n* = 2; lung cancer, *n* = 2; chronic obstructive pulmonary disease, *n* = 1; heart failure *n* = 1; tuberculosis, *n* = 1; unknown, *n* = 2).

### 3.7. MAC-PD-Associated Death

**Figure 1:**
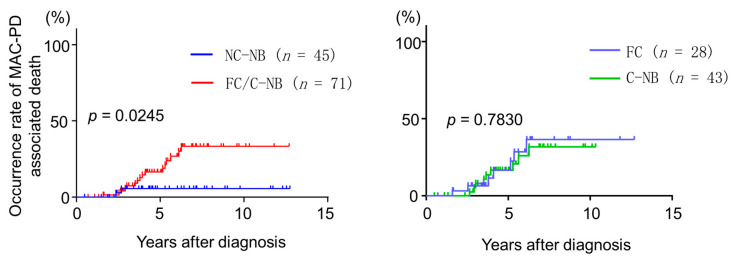
Occurrence rates of *Mycobacterium avium* complex pulmonary disease-associated death in patients with different forms of disease. The mortality rate of cavitary disease was high and different from that of non-cavitary disease. MAC-PD, *Mycobacterium avium* complex pulmonary disease; NC-NB, non-cavitary nodular bronchiectasis; FC, fibrocavitary form; C-NB, cavitary nodular bronchiectasis.

The occurrence rate of MAC-PD-associated death according to radiographic features was estimated (Figure 1). MAC-PD-associated death occurred mainly in patients with cavitary disease (88.9%). There was a significant difference in survival curves between patients with non-cavitary and cavitary NB (*p* = 0.0241). However, the survival curves for cavitary NB and FC disease were comparable (*p* = 0.7830). The 5-year occurrence rates of MAC-associated death for patients with cavitary NB disease and FC disease were 16.6% and 16.7%, respectively. We also analysed the prognostic factors in patients with cavitary disease (Table 5). Among the 75 patients with cavitary MAC-PD, univariate analysis identified age, low BMI, high C-reactive protein, diabetes mellitus, concurrent CPA, and acquired CAM resistance as risk factors for death. In contrast, standardized RFP + EB + CAM-based regimen and aminoglycoside use were significantly associated with positive outcome. Multiple logistic regression analysis revealed that age (*p* = 0.0136), diabetes mellitus (*p* = 0.041), and concurrent CPA (*p* = 0.0243) were independently associated with outcomes.

### 3.8. Occurrence Rate of CPA Diagnosis

**Figure 2:**
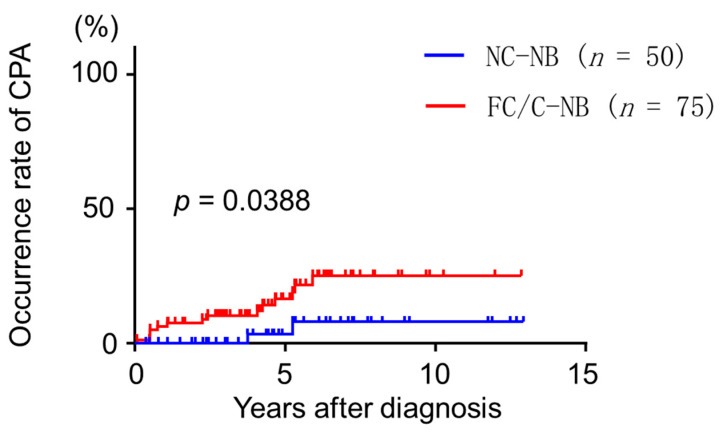
Occurrence rate of chronic pulmonary aspergillosis after diagnosis of *Mycobacterium avium* complex pulmonary disease. CPA, chronic pulmonary aspergillosis, NC-NB, non-cavitary nodular bronchiectasis; FC, fibrocavitary form; C-NB, cavitary nodular bronchiectasis.

**Figure 3:**
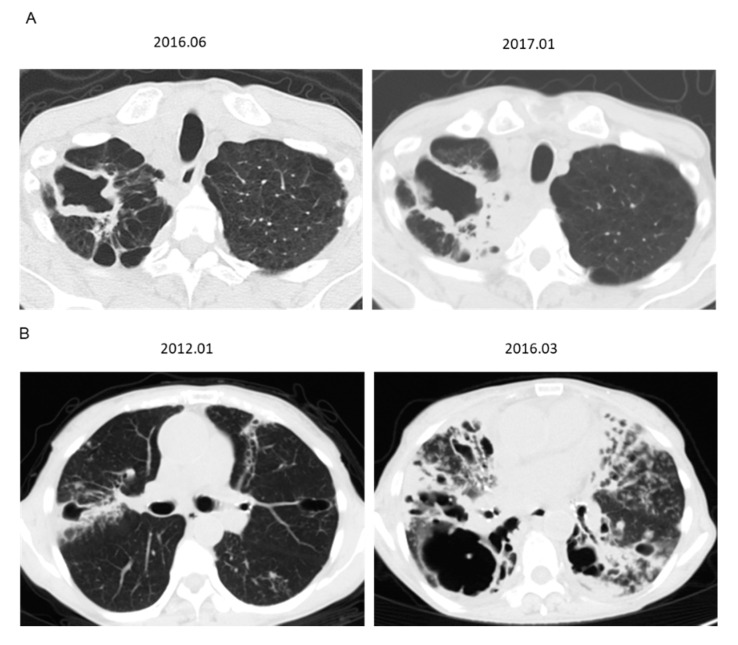
Serial computed tomography scans of patients with cavitary *Mycobacterium avium* complex pulmonary disease (MAC-PD) before and after diagnosis of chronic pulmonary aspergillosis (CPA). (**A**) Left: at the time of MAC-PD diagnosis (fibrocavitary form). *Aspergillus* galactomannan antigen 0.3, β-D glucan 8.8. Right: at the time of CPA diagnosis. *Aspergillus* galactomannan antigen 0.4, *Aspergillus* precipitating antibody-positive, β-D glucan 18.8 pg/mL, and isolation of *A. fumigatus* from sputum culture. (**B**) Left: at the time of MAC-PD diagnosis (cavitary nodular bronchiectasis). *Aspergillus* galactomannan antigen 0.2, β-D glucan 7.5. Right: at the time of CPA diagnosis. *Aspergillus* galactomannan antigen 5.0, *Aspergillus* precipitating antibody-positive, β-D glucan 300.0 pg/mL, and isolation of *A. fumigatus* from sputum culture.

CPA was diagnosed in 17 (13.6%) patients over a median follow-up period of 56.4 months (38.1–82.9) and the occurrence rate of CPA was estimated (Figure 2). There was a significant difference in CPA between patients with non-cavitary NB and cavitary NB/FC disease (*p* = 0.0388). The 5-year occurrence rates of CPA in patients with non-cavitary NB and cavitary NB/FC were 3.3 and 16.5%, respectively. Serial CT scans showed thickening of the cavitary wall with paracavity or extensive fibrosis (Figure 3).

## 4. Discussion

This study evaluated the prognosis and long-term outcome in 125 patients with chemo-naïve progressive MAC-PD hospitalized for the induction of multidrug combination therapy. In this study, the standardized RFP + EB + CAM-based regimen was shown to be an independent factor associated with negative-sputum conversion and a reduced rate of refractory disease, while aminoglycoside use was independently associated with a reduced rate of refractory disease.

Multidrug combination therapy is currently considered essential for preventing respiratory failure in patients with progressive MAC-PD, and RFP, EB, and macrolides are considered to be the standard multidrug regimen [6,13]. However, although macrolides are generally accepted as key drugs and a cornerstone of MAC treatment [14,15,16], no large studies have validated the appropriateness of current treatment guidelines for MAC-PD. Furthermore, even after successful treatment with antibiotic therapy, microbiological recurrence is relatively common [17,18]. However, most previous studies of combination therapy focused primarily on negative culture conversion with short-term observation periods [19,20,21], and the prognosis and long-term outcomes following combination therapy are thus poorly understood. In addition, FQs have been reported to be effective against MAC infection in vitro and in vivo and are widely and safely used, thus providing a promising alternative. However, data on the use of combination therapy including FQ are totally insufficient [22]. There is thus an urgent need to examine the efficacy and long-term outcomes of current standard regimens and other alternatives. The results of the current study are therefore important. In this study of 125 chemo-naïve patients with progressive MAC-PD, we showed that standardized regimens (RFP + EB + CAM containing at least three drugs), but not FQ-containing regimens, inhibited long-term disease progression, while the addition of an aminoglycoside inhibited refractory disease. In addition, although an FQ-containing three-drug regimen showed a comparable sputum-conversion rate to the RFP + EB + CAM regimen, the long-term disease-control rate was lower and CAM-resistant MAC strains were more frequent. This result coincides with a previous report that suggested the benefit of three-drug macrolide regimens including EB and RFP for protecting against macrolide resistance [23]. In contrast, a recent study showed that an EB + CAM-containing two-drug regimen was comparable to the standardized regimen (RFP + EB + CAM) in terms of the negative sputum-conversion rate [20]. However, the current results suggests that two-drug regimens should be selected cautiously in relation to long-term disease progression and persistence.

Numerous retrospective studies have reported on the prognostic factors of MAC-PD, and male sex, age, cavity, comorbidity, non-NB form, low BMI, and higher ESR have been associated with mortality [12,24,25]. *Aspergillus* coinfection was also reported in a small study involving patients with NTM-PD [26]. Furthermore, several issues remain to be clarified regarding MAC-PD disease type. The prognosis of cavitary NB has been suggested to differ from that of non-cavitary NB disease, rather than just being an advanced stage of the same disease [3]. However, many published studies failed to differentiate between these [20,27,28], and clarification of the prognostic difference between these two NB diseases is urgently needed to ensure the appropriate management of MAC-PD. This study, involving chemo-naïve 125 patients with progressive MAC-PD, showed that the risk of MAC-PD-associated death was higher in patients with cavitary disease, and age, diabetes mellitus, and CPA were independently associated with mortality among patients with cavitary MAC-PD. We also showed that mortality was significantly lower in patients with non-cavitary NB disease compared with patients with cavitary NB and FC disease. No patients changed from non-cavitary to cavitary NB in this study. These results suggested that cavitary disease, including cavitary NB, may be completely different from non-cavitary NB, rather than just an advanced stage. Indeed, fewer than 5% of patients with non-cavitary disease experienced MAC-PD-associated death, compared with about 25% of patients with cavitary MAC-PD, despite intensive antimicrobial therapy. However, we cannot exclude the possibility that the apparent lack of progression to cavitary disease may be ascribed to the effects of combination antimicrobial treatment. Future studies of the natural history of NB disease are necessary to confirm if cavitary NB disease is totally different from non-cavitary NB disease.

The incidence of refractory cases increased during the observation period, despite the use of multidrug combination therapy, finally accounting for almost half of all patients (45.6% in this study). The treatment of refractory MAC-PD presents a major clinical problem, requiring a thorough analysis of the factors associated with this condition. The current study revealed that BMI and acquired CAM resistance were independent risk factors for refractory disease. Notably, 21 (16.8%) patients acquired CAM resistance during the observation period, which led to poor treatment outcomes [23]. We also showed that use of a standardized RFP + EB + CAM-based regimen and the addition of an aminoglycoside inhibited future disease persistence. In contrast, FQ-containing regimens induced a significantly higher rate of acquired CAM resistance.

Risk factor analysis for mortality identified age, BMI, chronic systemic inflammation, and diabetes mellitus, which lead to reduced immune regulation, and cavitary form of the disease, which is a reflection of pulmonary parenchymal damage caused by MAC, as factors significantly associated with death. These factors predispose MAC-PD patients to the development of chronic Aspergillus infection, which is a severely debilitating disease often resulting in a shortened lifespan [29]. As a consequence, CPA was also an independent risk factor for death, especially in patients with cavitary MAC-PD. Overall, 17.3% of patients with cavitary MAC-PD were diagnosed with CPA, mainly 2–5 years after a MAC diagnosis, and 61.5% died during the observation period. The high incidence and significant mortality of CPA in patients with cavitary MAC-PD suggests the need for prophylactic measures in future practice.

Azoles such as voriconazole and itraconazole are important for the treatment of CPA, but the interactions of these drugs with RFP are well known. Interactions between such antifungal agents and RFP makes it difficult to select suitable therapeutic drugs, which, together with diagnostic difficulties, may contribute to the poor prognosis of MAC-PD patients complicated with CPA. Among 17 CPA patients, RFP was prescribed to eight patients at the diagnosis of CPA. Among these eight patients, two patients stopped RFP because of concerns regarding drug interactions, micafungin (MCFG) was used in five patients, and itraconazole (ITZ) and voriconazole (VRCZ) were used in one patient each. Among the other nine patients, caspofungin (CPFG) was used in three patients, MCFG in five, ITZ in two, VRCZ in one, and liposomal amphotericin B (L-AMB) in one patient. We did not check drug levels of antifungal agents. Interruption of RFP may result in inadequate MAC treatment, and continuation of RFP with avoidance of ITZ and VRCZ in light of drug interactions may result in inadequate CPA treatment, both of which could lead to a poor prognostic outcome. Difficulties in assessing the disease activity and severity of CPA in MAC-PD patients meant that intermittent administration of candins, such as CPFG and MCFG, was the main therapy in this study, and long-term oral anti-fungal agents were administered in less than a third of CPA patients (29.4%). Thus, considering the high frequency and mortality of this complication, treatment strategies for CPA and MAC co-infection need to be examined and determined.

Furthermore, MAC-PD and fungal PD in adult bronchiectasis patients are associated with airway clearance defects [30]. All patients with MAC-PD would therefore benefit from airway-clearance therapies, in addition to optimal combinations of antibiotics. Considering the high rate of refractory disease despite the use of multidrug combination therapy, airway-clearance therapy would improve the conversion rate to sputum negativity, reduce the relapse frequency, and reduce disease persistence and progression.

The current study had some limitations. First, it was a retrospective study and we therefore could not exclude potential confounding factors, such as microbiologic and other laboratory data. Second, this study included a heterogeneous population of progressive MAC-PD patients hospitalized for initial combination antimicrobial drug therapy. Given that patients who are hospitalized in a referral centre for first-line combination treatment tend to have advanced disease, this might have affected the clinical outcome following treatment, and these results therefore cannot be generally applied to all MAC-PD patients undergoing combination therapy. Prospective large-scale studies are therefore needed to evaluate the outcomes of first-line combination therapies, involving strict statistics and unbiased methods. Third, patients were only selected from one referral centre with experience in the management of MAC-PD, which might have led to selection bias. Finally, we did not evaluate the factors affecting decision-making by pulmonary physicians with regard to the timing and selection of antimicrobial therapy. Indeed, of 39 patients treated with a modified regimen, 12 were initially treated with a standardized regimen, which was modified soon after the start of treatment because of side effects (RFP: exanthema, *n* = 3, fever, *n* = 3, hepatotoxicity, *n* = 2; EB: visual impairment, *n* = 2; CAM: exanthema, *n* = 1, nausea + abdominal pain, *n* = 1). On the other hand, one patient treated with a standardized regimen developed exanthema and fever caused by RFP and underwent successful drug desensitization therapy, one patient stopped EB because of visual impairment more than 1 year after initiation of standard treatment, and one patient continued standardized treatment despite CAM-induced exanthema. Finally, no patients in this study were diagnosed with cystic fibrosis, although examinations to definitely exclude this disease, such as *CFTR* polymorphisms, deltaF508 heterozygosity, and sweat chloride tests, were not performed. However, a previous study reported that the incidence of cystic fibrosis in the Japanese population was much lower than other countries, and all the patients in the current study were Japanese, with an average age of 68 years at diagnosis (60–73.5 years) [31]. We therefore considered it unlikely that the lack of definite exclusion of cystic fibrosis would have affected the results of this study. However, a previous study in the United States involving adult patients with bronchiectasis, including MAC-PD, showed a significantly elevated prevalence of *CFTR* mutations when they were screened for an expanded range of polymorphisms. Further research applying clinical and genomic criteria based on our current knowledge of cystic fibrosis is therefore necessary to clarify the incidence of cystic fibrosis in Japan [32].

## 5. Conclusions

In conclusion, the present study clarified that the choice of first-line treatment was independently associated with sputum conversion and the development of refractory disease in patients with progressive MAC-PD. The use of an RFP + EB + CAM-based regimen was significantly and independently associated with negative sputum conversion and reduced disease progression, although the side effects were not acceptable in some patients. Aminoglycoside addition reduced the risk of refractory disease. Regarding the prognosis, cavitary NB was associated with a higher mortality than non-cavitary disease, and seems to be a totally different disease, rather than just an advanced form of non-cavitary NB. CPA was prevalent among patients with cavitary MAC-PD and was independently associated with increased mortality. Host factors affecting systemic immune regulation, and cavitary lesions reflecting pulmonary parenchymal damage, predispose MAC-PD patients to the development of chronic devastating Aspergillus infection. Intensive multidrug combination therapy including aminoglycosides should be administered to patients with progressive MAC-PD to inhibit disease progression and persistence. However, these results were based on patients with progressive MAC-PD hospitalized for the induction of first-line combination therapy, and further prospective, large-scale studies are therefore needed to validate these promising results.

## Figures and Tables

**Table 1:** Treatment regimen.

Treatment Regimen (*n* = 125)	No. (%)
RFP + EB + CAM	82 (65.6)
RFP + EB + CAM + FQ	4 (3.2)
EB + CAM + FQ	10 (8.0)
RFP + CAM + FQ	7 (5.6)
CAM + FQ	4 (3.2)
EB + FQ	1 (0.8)
EB + CAM	11 (8.8)
RFP + CAM	4 (3.2)
RFP + EB	2 (1.6)

RFP, rifampicin; EB, ethambutol; CAM, clarithromycin; FQ, fluoroquinolones.

**Table 2:** Baseline characteristics and analysis of sputum conversion in patients with *Mycobacterium avium* complex pulmonary disease, no. (%) or median (IQR).

				Univariate Analysis		Multivariate Analysis	
	Total (*n* = 125)	Conversion (*n* = 100)	Non-Conversion (*n* = 25)	*p*-Value	OR (95%CI)	*p*-Value	Adjusted OR (95%CI)
Characteristic							
Sex, female	85 (68.0)	73 (73.0)	12 (48.0)	0.0292	0.34 (0.14–0.80)	0.0756	
Age, years	68 (60–73.5)	68 (60–73)	69 (63.5–75)	0.278		0.4364	
BMI	17.46 (16.16–19.77)	17.56(16.32–20.3)	17.1 (15.9–19)	0.219		0.4012	
Underlying disease							
COPD	5 (4.0)	3 (3.0)	2 (8.0)	0.261			
Old Tb	11 (8.8)	7 (7.0)	4 (16.0)	0.228			
DM	8 (6.4)	2 (2.0)	6 (24.0)	0.0008	15.47(3.40–77.25)	0.0586	
CRP	0.38 (0.1–2.26)	0.225 (0.1–1.05)	2.13(0.81–4.88)	<0.0001		0.1424	
NB form	89 (71.2)	70 (70.0)	19 (76.0)	0.629			
Cavitation	80 (64.0)	63 (63.0)	17 (68.0)	0.816		0.7808	
*M. avium*	71 (56.8)	57 (57.0)	14 (56.0)	>0.999			
Treatment duration, days	450 (294.5–825.5)	447.5 (345–756.3)	547(167.5–1266)	0.8697			
RFP + EB + CAM-containing regimen	86 (68.8)	75 (75.0)	11 (44.0)	0.0068	0.26 (0.10–0.63)	0.0238	0.298 (0.11–0.85)
FQ-containing regimen	25 (20.0)	19 (19.0)	6 (24.0)	0.5878			
Aminoglycoside use	53 (42.4)	47 (47.0)	6 (24.0)	0.0434	0.35 (0.13–0.98)	0.2158	

IQR, interquartile range; OR, odds ratio; CI, confidence interval; BMI, body mass index; COPD, chronic obstructive pulmonary disease; Tb, tuberculosis; DM, diabetes mellitus; CRP, C-reactive protein; NB, nodular bronchiectasis; RFP + EB + CAM, rifampicin + ethambutol + clarithromycin; FQ, fluoroquinolones.

**Table 3:** Treatment selection modified disease progression and persistence in patients with *Mycobacterium avium* complex pulmonary disease.

			Univariate Analysis		Multivariate Analysis	
	Treatment Success (*n* = 68)	Refractory Disease ^a^ (*n* = 57)	*p*-Value	OR (95%CI)	*p*-Value	Adjusted OR (95%CI)
Characteristic						
Sex, female	50 (73.5)	35 (61.4)	0.1792		0.0599	
Age, years	67 (60.5–72)	69 (60–74.5)	0.3995		0.3091	
BMI	18.25 (16.46–21.03)	17.05 (16.07–18.77)	0.0198		0.0162	
DM	5 (7.4)	7 (12.3)	0.3783			
CRP	0.27 (0.1–1)	0.64 (0.1–2.873)	0.1147			
NB form	46 (67.6)	43 (75.4)	0.4283			
Cavitation	42 (61.8)	38 (66.7)	0.5813		0.8641	
*M. avium*	36 (52.9)	35 (61.4)	0.7112			
Treatment duration, days	441 (351.3–694.5)	459 (220.5–1184)	0.5182			
RFP + EB + CAM-containing regimen	57 (83.8)	29 (50.9)	<0.0001	0.199 (0.091–0.457)	0.0023	0.244 (0.098–0.605)
FQ-containing regimen	12 (17.6)	13 (22.8)	0.8252			
Aminoglycoside use	39 (57.4)	14 (24.6)	0.0003	0.242 (0.114–0.537)	0.0056	0.304 (0.131–0.705)
Acquired condition						
CAM resistance	2 (2.9)	19 (33.3)	<0.0001	16.5 (4.23–73.37)	0.0002	20.12 (4.13–97.94)
Concurrent CPA	6 (8.8)	11 (19.3)	0.1171			

Values given as no. (%) or median (IQR). ^a^ Refractory case: no sputum conversion, or recurrence and persistent positive sputum status until the end of follow-up. OR, odds ratio; CI, confidence interval; IQR, interquartile range; BMI, body mass index; CPA, chronic pulmonary aspergillosis.

**Table 4:** Comparison of RFP + EB + CAM and RFP/EB + CAM + FQ regimens.

			Univariate Analysis	
	RFP + EB + CAM (*n* = 82)	RFP/EB + CAM + FQ (*n* = 17)	*p*-Value	OR (95%CI)
Characteristic				
Sex, female	54 (64.9)	12 (70.6)	0.7845	
Age, years	69 (60–73)	70 (64.5–75.5)	0.3794	
BMI	17.56(16.61–20.11)	17.46 (16.1–19.93)	0.8433	
DM	8 (9.8)	1 (5.9)	>0.9999	
CRP	0.32 (0.1–1.93)	0.18 (0.1–3.505)	0.9702	
NB form	59 (72.0)	15 (88.2)	0.2248	
Cavitation	50 (61.0)	11 (64.7)	>0.9999	
*M. avium*	43 (52.4)	12 (70.6)	0.1921	
Treatment duration, days	441 (351.3–694.5)	459 (220.5–1184)	0.5182	
Aminoglycoside use	42 (51.2)	5 (29.4)	0.1171	
Sputum conversion	71 (86.6)	13 (76.5)	0.2831	
Refractory case	26 (31.7)	11 (64.7)	0.0141	3.95 (1.40–12.2)
Acquired condition				
CAM resistance	7 (8.5)	7 (41.2)	0.0022	7.5 (2.16–26.9)
Concurrent CPA	9 (11.0)	3 (17.6)	0.1171	0.4277

Values given as no. (%) or median (IQR). OR, odds ratio; CI, confidence interval; BMI, body mass index; DM, diabetes mellitus; CRP, C-reactive protein; NB, nodular bronchiectasis; IQR, interquartile range; RFP + EB + CAM, rifampicin + ethambutol + clarithromycin; FQ, fluoroquinolone; CAM, clarithromycin; CPA, chronic pulmonary aspergillosis.

**Table 5:** Risk factor analysis in patients with cavitary *Mycobacterium avium* complex pulmonary disease.

			Univariate Analysis		Multivariate Analysis	
	Censored (*n* = 59)	MAC-PD-Associated Death (*n* = 16)	*p*-Value	OR (95%CI)	*p*-Value	Adjusted OR (95%CI)
Characteristic						
Sex, female	41 (69.5)	7 (43.8)	0.079		0.23	
Age, years	67 (58–71)	75 (69.5–77)	0.0002		0.033	
BMI	17.56 (16.77–20)	16.24 (14.9–18.83)	0.0150		0.084	
DM	2 (3.4)	4 (25.0)	0.0168	9.5 (1.94–52.01)	0.041	9.96 (1.10–90.09)
CRP	0.38 (0.1–1.463)	2.73 (1.09–5.46)	0.0021		0.82	
NB form	34 (57.6)	9 (56.3)	>0.9999			
*M. avium*	31 (52.5)	8 (50.0)	>0.9999			
Treatment duration, days	589 (387–1325)	476 (143.5–745.5)	0.1257			
RFP + EB + CAM-containing regimen	44 (74.6)	6 (37.5)	0.0079	0.2045 (0.062–0.67)	0.66	
FQ-containing regimen	12 (20.3)	5 (31.3)	0.501			
Aminoglycoside use	28 (47.5)	3 (18.8)	0.0478	0.2555 (0.072–0.96)	0.82	
Acquired condition						
CAM resistance	9 (15.3)	7 (43.8)	0.0335	4.321 (1.28–14.11)	0.115	
Concurrent CPA	5 (8.5)	8 (50.0)	0.0006	10.8 (2.84–40.94)	0.0235	8.552 (1.335–54.77)

MAC-PD, *Mycobacterium avium* complex pulmonary disease; OR, odds ratio; CI, confidence interval; BMI, body mass index; DM, diabetes mellitus; CRP, C-reactive protein; NB, nodular bronchiectasis; RFP + EB + CAM, rifampicin + ethambutol + clarithromycin; FQ, fluoroquinolone; CAM, clarithromycin; CPA, chronic pulmonary aspergillosis.

## Supplementary Materials

The following are available online at https://www.mdpi.com/2077-0383/9/5/1315/s1, Figure S1: Study design, Figure S2: Analysis of MAC-PD associated death.

## Abbreviations

BMI	body mass index
CAM	clarithromycin
CPA	chronic pulmonary aspergillosis
CT	computed tomography
EB	ethambutol
FC	fibrocavitary
FQ	fluoroquinolones
HIV	human immunodeficiency virus
MAC	*Mycobacterium avium* complex
MAC-PD	*Mycobacterium avium* complex pulmonary disease
NB	nodular bronchiectasis
NTM-PD	non-tuberculous mycobacterial pulmonary disease
RFP	rifampicin
