# Supplementary material for: Long-Term Treatment Outcome of Progressive Mycobacterium avium Complex Pulmonary Disease
Source: J Clin Med. 2020 May 2;9(5):1315. doi: 10.3390/jcm9051315 (PMC7291046; doi:10.3390/jcm9051315)
Supplement: Supplementary file 1 [file jcm-09-01315-s001.pdf]

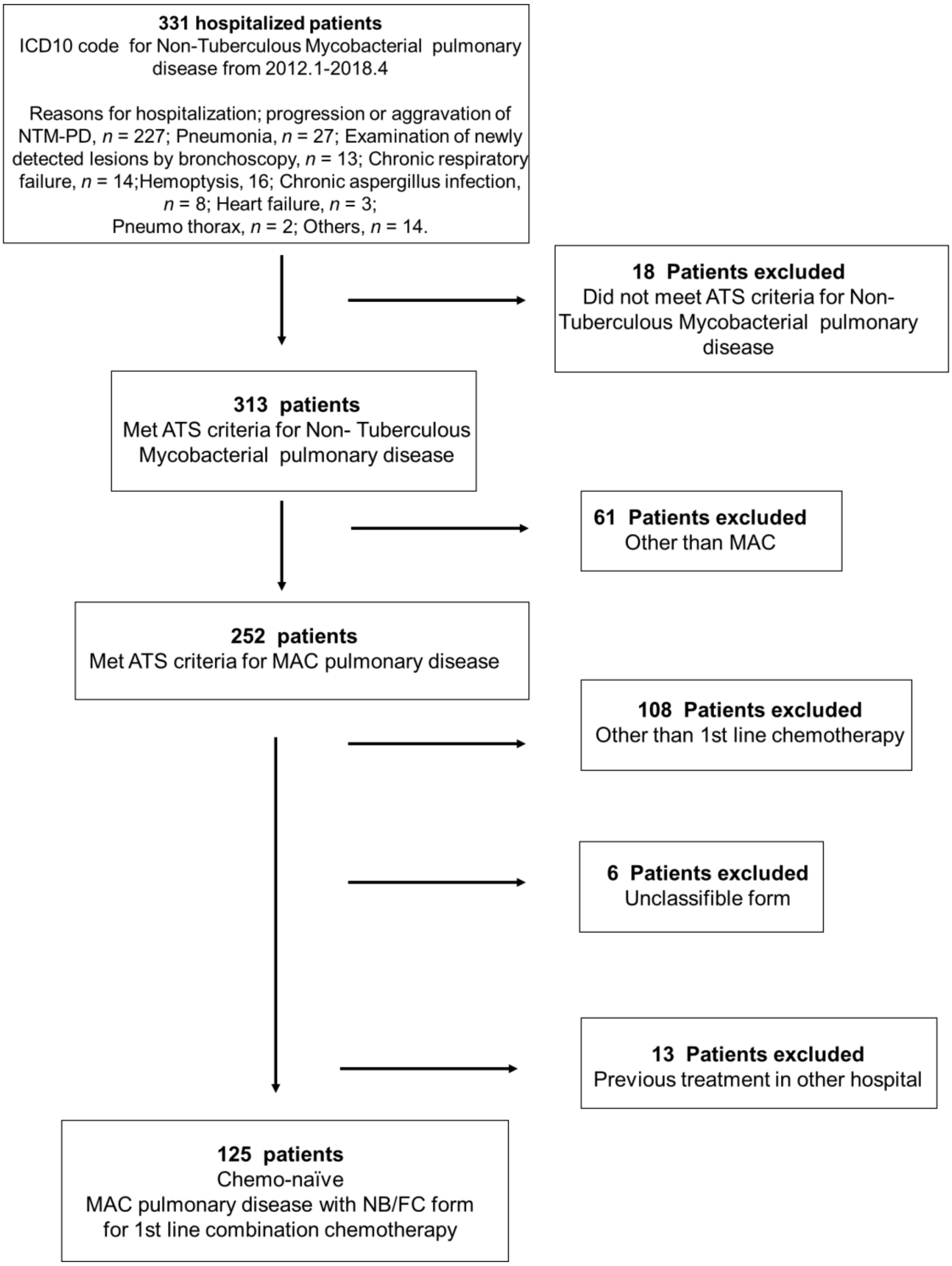

Figure S1. Study design. ICD 10, international classification of disease 10; ATS, American Thoracic Society; NTM-PD; non-tuberculous mycobacterial pulmonary disease; MAC, *Mycobacterium avium* complex; NB, nodular bronchiectasis; FC, fibrocavitary.

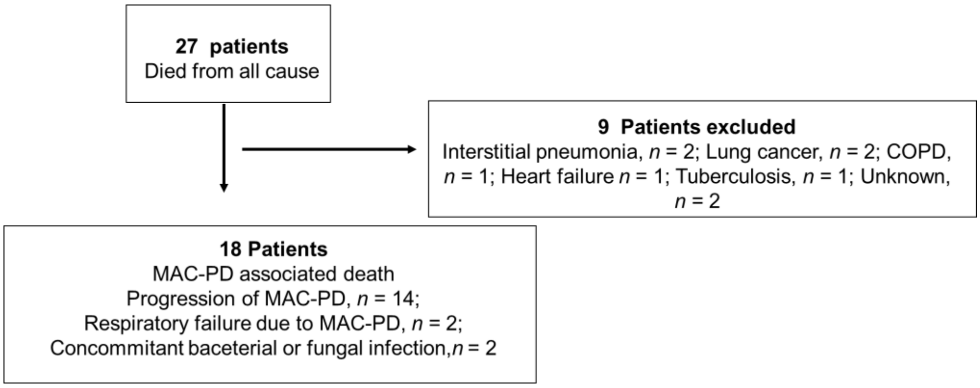

Figure S2. Analysis of MAC-PD associated death. COPD, chronic obstructive pulmonary disease; MAC-PD, *Mycobacterium avium* complex pulmonary disease.
